# Conversion of Ultrasmall Glutathione-Coated Silver Nanoparticles during Dispersion in Water into Ultrasmall Silver Sulfide Nanoparticles

**DOI:** 10.3390/nano14171449

**Published:** 2024-09-05

**Authors:** Natalie Wolff, Oleg Prymak, Nataniel Białas, Torsten Schaller, Kateryna Loza, Felix Niemeyer, Marc Heggen, Claudia Weidenthaler, Cristiano L. P. Oliveira, Matthias Epple

**Affiliations:** 1 Inorganic Chemistry and Centre for Nanointegration Duisburg-Essen (CENIDE), University of Duisburg-Essen, Universitaetsstr. 5-7, 45117 Essen, Germany; natalie.wolff@uni-due.de (N.W.); oleg.prymak@uni-due.de (O.P.); nataniel.bialas@uni-due.de (N.B.); kateryna.loza@uni-due.de (K.L.); 2 Organic Chemistry, University of Duisburg-Essen, Universitaetsstr. 5-7, 45117 Essen, Germany; torsten.schaller@uni-due.de (T.S.); felix.niemeyer@uni-due.de (F.N.); 3 Ernst Ruska Centre for Microscopy and Spectroscopy with Electrons, Forschungszentrum Jülich, 52428 Jülich, Germany; m.heggen@fz-juelich.de; 4 Max-Planck-Institut für Kohlenforschung, 45470 Mülheim an der Ruhr, Germany; weidenthaler@mpi-muelheim.mpg.de; 5 Institute of Physics, University of São Paulo, São Paulo 05508-090, Brazil; crislpo@if.usp.br

**Keywords:** silver, nanoparticles, NMR spectroscopy, nanoclusters, ultrasmall, glutathione

## Abstract

Ultrasmall silver nanoparticles (2 nm) were prepared by reduction with sodium borohydride (NaBH_4_) and stabilized by the ligand glutathione (a tripeptide: glycine–cysteine–glutamic acid). NMR spectroscopy and optical spectroscopy (UV and fluorescence) revealed that these particles initially consist of silver nanoparticles and fluorescing silver nanoclusters, both stabilized by glutathione. Over time, the silver nanoclusters disappear and only the silver nanoparticles remain. Furthermore, the capping ligand glutathione eliminates hydrogen sulfide (H_2_S) from the central cysteine and is released from the nanoparticle surface as tripeptide glycine–dehydroalanine–glutamic acid. Hydrogen sulfide reacts with the silver core to form silver sulfide. After four weeks in dispersion at 4 °C, this process is completed. These processes cannot be detected by transmission electron microscopy (TEM), small-angle X-ray scattering (SAXS), or differential centrifugal sedimentation (DCS) as these methods cannot resolve the mixture of nanoparticles and nanoclusters or the nature of the nanoparticle core. X-ray photoelectron spectroscopy showed the mostly oxidized state of the silver nanoparticle core, Ag(+I), both in freshly prepared and in aged silver nanoparticles. These results demonstrate that ultrasmall nanoparticles can undergo unnoticed changes that considerably affect their chemical, physical, and biological properties. In particular, freshly prepared ultrasmall silver nanoparticles are much more toxic against cells and bacteria than aged particles because of the presence of the silver clusters.

## 1. Introduction

Ultrasmall nanoparticles have a diameter of 1–3 nm and are at the border of atomically precise metal clusters [1,2]. They are typically prepared by reducing the corresponding metal salts with a strong reducing agent like NaBH_4_ in the presence of a coating agent, e.g., a cysteine-containing peptide or a thiolated molecule [3,4]. Their very high specific surface area offers the potential to attach functional ligands with a high surface density [5,6]. This is enhanced by the high surface curvature so that the ligand density is higher than on a planar metal surface of larger (e.g., plasmonic) nanoparticles. This corresponds to a low molecular footprint of about 0.1 nm^2^ for short peptides [7].

As it is well known from heterogeneous catalysis, small particles are particularly reactive because of their high specific surface area and their high surface curvature that exposes many crystal faces and causes local defects [8,9,10,11,12]. Thermodynamically, this is mirrored by a high surface energy, shifting small particles upwards in the free enthalpy of formation. Ultrasmall silver nanoparticles have gained special attention because they may exert a higher bactericidal effect than larger silver nanoparticles [13,14,15].

The analysis of ultrasmall particles is challenging. Classical methods like scanning electron microscopy and dynamic light scattering are not applicable because the particles are so small [5,6]. Conventional X-ray powder diffraction is a challenge because small particles lead to considerably broadened diffraction peaks [16,17]. Therefore, high-resolution transmission electron microscopy (HRTEM) as well as small-angle X-ray scattering (SAXS) are the methods of choice. However, it is also possible to apply solution NMR spectroscopy on dispersed ultrasmall nanoparticles because their influence on the NMR signal of the attached ligands is tolerable. This can give valuable insight into the chemical nature of the ligand shell that is not available from HRTEM or SAXS [18,19,20].

Ultrasmall nanoparticles of several metals have been investigated [6,13,21,22,23,24]. To avoid oxidation by the solvent water and dissolved oxygen, only noble metals can be used. The classical example is gold, for which a broad array of ultrasmall nanoparticles and atomically precise clusters are known [11,21,22,25,26,27]. In-depth studies have shown that the core consists of metallic gold, usually covered with thiolated ligands that form a stable Au-S bond [28]. There is no indication that ultrasmall gold nanoparticles undergo oxidation with time, i.e., they remain metallic in nature [16].

In contrast, it has been demonstrated that ultrasmall nanoparticles of silver and platinum metals are partially or fully oxidized [16,17]. This was mainly derived from X-ray photoelectron spectroscopy (XPS) which gives the oxidation state of the metal. Supporting information came from X-ray powder diffraction and from electron diffraction. However, radiation damage cannot be excluded in transmission electron microscopy, especially for ultrasmall nanoparticles [29]. Notably, structural changes that easily can escape attention have also been reported for metal clusters in heterogeneous catalysis [30,31].

Here, we demonstrate that ultrasmall glutathione (GSH)-capped silver nanoparticles change upon storage in dispersion. A freshly prepared sample consists of a mixture of silver nanoparticles and smaller silver nanoclusters. After about four weeks in dispersion, the silver nanoclusters disappear and only the nanoparticles are left behind. The capping ligand glutathione is bound to the metal surface via the thiol group of cysteine. Over time, the central cysteine in glutathione eliminates hydrogen sulfide to form dehydroalanine. The resulting tripeptide glycine–dehydroalanine–glutamic acid is released into the solution. Hydrogen sulfide reacts with the silver metal core to form silver sulfide.

These effects are important because the toxicity of the silver nanoparticles towards eukaryotic cells and bacteria changes during storage. Thus, an in-depth characterization of such systems is necessary to avoid wrong conclusions.

## 2. Materials and Methods

### 2.1. Chemicals and Reagents

Silver nitrate (AgNO_3_, 99%) was obtained from Carl Roth (Karlsruhe, Germany). Nitric acid (HNO_3_, 67%), hydrochloric acid (HCl, 37%), and sodium hydroxide (NaOH, 1 M) were obtained from Bernd Kraft (Duisburg, Germany). Sodium borohydride (NaBH_4_, >96%) and 10 kDa spin filters were obtained from Merck (Darmstadt, Germany). Glutathione (GSH, 98%) and maleic acid (99%) were obtained from Sigma-Aldrich (Steinheim, Germany). Deuterium oxide (D_2_O, 99.9%) was obtained from Deutero GmbH (Kastellaun, Germany). L(-)-Glutathione disulfide (GSSG, 95%), dodecane (99%), and 2-(4,5-dimethylthiazol-2-yl)-3,5-diphenyl-2H-tetrazol-3-ium bromide (MTT) were obtained from Thermo Fisher Scientific (Schwerte, Germany). Gibco™ FBS, Gibco™ DMEM, Gibco™ sodium pyruvate, Gibco™ GlutaMAX, Gibco™ Trypsin-EDTA, Gibco™ DPBS, and Gibco™ penicillin–streptomycin were obtained from ThermoFisher Scientific (Waltham, MA, USA). HeLa cells were obtained from the American Type Culture Collection (ATCC, Manassas, VA, USA). Ultrapure water (ELGA Purelab, ELGA LabWater, Celle, Germany) was used for all syntheses and analyses unless stated otherwise.

### 2.2. Methods

The silver concentration was determined by atomic absorption spectroscopy (AAS) with a Thermo Electron M-Series spectrometer (graphite tube furnace). Briefly, 5 μL of a nanoparticle dispersion was dissolved in concentrated nitric acid (955 μL) and diluted with 3 mL water.

UV/Vis spectroscopy was performed with a Genesis 50 instrument (ThermoScientific, Waltham, MA, USA) in quartz glass cuvettes in the range of 200 to 800 nm.

Fluorescence spectroscopy was performed with a Cary Eclipse spectrometer (Agilent Technologies, Santa Clara, CA, USA) in a fluorescence cuvette (600 µL). The excitation spectrum of the sample was recorded to determine the most appropriate excitation wavelength for fluorescence. This was followed by the measurement of an emission spectrum.

Differential centrifugal sedimentation (DCS) was performed with a DC 24000 instrument (CPS Instruments, Prairieville, LA, USA). A density gradient was generated with sucrose solutions (8 and 24 wt%), and 0.5 mL dodecane was used as a stabilizing agent to prevent evaporation. Polyvinyl chloride particles with a defined size of 483 nm provided by CPS Instruments were used as a calibration standard. A density of 10,490 kg m^−3^ was used for the freshly prepared nanoparticles (metallic silver) and 7230 kg m^−3^ for the aged nanoparticles (silver sulfide).

The samples were analyzed by aberration-corrected scanning transmission electron microscopy to verify the aging of nanoparticles. The nanoparticles were suspended in water and deposited on an ultrathin amorphous carbon Cu TEM grid by drop-casting. The water was allowed to evaporate in the air. Following this, the samples were cleaned with a UV-based sample cleaner (Hitachi HT ZONETEM II, Tokyo, Japan). High-resolution imaging was performed on a Hitachi HF5000 at 200 kV, equipped with a spherical-aberration (Cs) probe corrector.

For NMR spectroscopy, the nanoparticles were dispersed in 540 µL H_2_O and measured after mixing with 60 µL D_2_O with water suppression at pH 8.5. The peptides GSH and GSSG were measured in pure D_2_O at pH 8.5. NMR spectra were recorded with Bruker Avance Neo 400 MHz and Bruker Avance III 600 MHz spectrometers.

X-ray powder diffraction (XRD) was performed with a Bruker D8 Advance diffractometer (Bruker, Billerica, MA, USA), with Cu Kα radiation (*λ* = 1.54 Å) operating at 40 kV and 40 mA. A dispersion of nanoparticles was placed on a silicon single-crystal sample holder to minimize scattering and dried in air. The samples were measured in reflection mode from 20 to 90° 2*θ* with a step size of 0.02° and a counting time of 8 s per step. Qualitative phase analysis was performed with Diffrac.Suite EVA V1.2 from Bruker with the patterns of the metals Ag (#04-0783) and silver sulfide Ag_2_S (#24-0715; acanthite) from the ICDD database.

X-ray photoelectron spectroscopy (XPS) was performed with a spectrometer from SPECS GmbH (Berlin, Germany), equipped with a Phoibos 150 1D-DLD hemispherical energy analyzer. The monochromatized Al Kα X-ray source (*E* = 1486.6 eV) was operated at 15 kV and 200 W. For high-resolution scans, the pass energy was set to 20 eV, and the medium area mode was used as lens mode. The base pressure in the analysis chamber was 4 × 10^−10^ mbar during the experiment. To account for charging effects, all spectra referred to C 1s at 284.5 eV.

Small-angle X-ray scattering was carried out at the EMUSAXS center located at the Institute of Physics, University of São Paulo. The laboratory-based equipment Xeuss2.0 (Xenocs SAS, Grenoble, France) was used for data acquisition. This machine was equipped with a microfocus Genix3D (Xenocs SAS, Grenoble, France) Cu Kα source (*λ* = 1.54 Å), Fox3D mirrors, and two sets of scatterless slits. Data acquisition was performed with a 2D pixel detector Dectris-Pilatus 300k (Baden, Switzerland). The integration of the images was performed by the program Fit2D [32]. As a result, 1D curves of the scattering intensity as a function of the momentum transfer modulus *q* were obtained. *q* is defined as *q* = 4 π sin(*θ*)/*λ*, where *θ* is the scattering angle. Data treatment and error propagation were performed with the program package SuperSAXS [33]. A sample-to-detector distance of 510 mm was used, leading to an available range of 0.012 < *q* < 0.71 Å^−1^.

Samples were analyzed both in dispersion and as a freeze-dried powder. Powder samples were sandwiched between two mica windows, with the empty mica windows measured as blank scattering for later data treatment. For the dispersed nanoparticles, reusable sample holders composed of borosilicate glass capillaries glued on stainless steel cases and closed with rubber caps were used. In all cases, the measurements were performed in vacuum to decrease the background scattering from air. The obtained SAXS data were evaluated with a model composed of polydisperse spheres (radius *R*, polydispersity *σ*) with hard-sphere interactions (volume fraction *η*, interaction radius, *R*_HS_). For the powder samples, it was necessary to include additional structure factors to describe the aggregation. After several tests, a structure factor of a fractal aggregate [34] (fractal dimension *D*_FR_, domain size *ζ*, subunit radius *R*_0_, and scale of the structure factor, *SC_FR_*) was chosen to describe the scattering data.

### 2.3. Synthesis of Silver Nanoparticles

The particles were synthesized by a modified Brust–Schiffrin synthesis [3,4,35,36]. An improved version of a synthesis reported earlier [16,37] was used to prepare Ag-GSH nanoparticles [16]. A 250 mL round-bottomed flask was filled with 90 mL water and degassed with argon for 15 min. Then, 101.9 mg silver nitrate (600 µmol, 64.7 mg Ag, 1 eq.) and 553.2 mg glutathione (1.8 mmol, 3 eq.) were dissolved in 1 mL of water and added. The white turbid dispersion was stirred at 0 °C for 30 min. NaBH_4_ (227 mg, 6 mmol, 10 eq.) was dissolved in 5 mL of ice-cold water and quickly added to the dispersion. The clear orange dispersion was warmed to room temperature and stirred for 1 h, after which it turned dark red. Nanoparticles were isolated by spin filtration and washed twice with 0.1 M NaOH and six times with water (10 kDa Amicon spin filters at 4000 rpm, 2500 g, 20 min) to remove unbound GSH and synthesis by-products. The yield of silver as determined by AAS was 88% (57 mg Ag). For aging, the particles were kept in dispersion (unstirred) at 4 °C. Neither precipitation nor agglomeration were observed.

### 2.4. MTT Tests of Nanoparticles with HeLa Cells

The viability after nanoparticle incubation was determined with an MTT assay using HeLa cells. HeLa cells were seeded at a density of 20,000 cells per well in a 24-well plate and incubated with 0.5 mL DMEM for 12 h at 37 °C and 5% CO_2_ atmosphere. Next, the cells were incubated with silver nanoparticles. The nanoparticles were tested at metal concentrations between 2.5 and 100 µg mL^−1^. Untreated HeLa cells cultured in a medium served as control. After incubation for 24 h, the cells were washed three times with DPBS to remove adherent and dispersed nanoparticles. To prepare the staining solution, 30 mg MTT was dissolved in 5 mL DPBS and diluted with 25 mL DMEM to the final concentration of 1 mg mL^−1^. To each well, 0.3 mL staining solution was added. The cells were incubated for 1 h at 37 °C in 5% CO_2_ atmosphere. The solution was removed, and 0.3 mL DMSO was added to each well. The cells were incubated for 30 min at room temperature. The concentration of dissolved formazan was quantified in a 96-well plate with a Multiscan plate reader (Thermo Fisher Scientific GmbH) at 570 nm. For each nanoparticle concentration, at least two independent cell culture experiments were performed.

### 2.5. Antibacterial Tests of Nanoparticles with Staphylococcus xylosus and Escherichia coli

The minimal inhibitory concentration (MIC) is defined as the lowest concentration of an antimicrobial agent or drug that inhibits the growth of a given microbial strain in vitro. Bacterial strains were cultivated in Lysogeny Broth (LB, 20 g L^−1^) and Trypticase Soy Broth (TSB). TSB was prepared by mixing CASO Bouillon (30 g L^−1^) and yeast extract (3 g L^−1^). To prepare solid media, Agar-Agar Kobe I was added to liquid media (15 g L^−1^). All reagents for media preparation were obtained from Carl Roth, Karlsruhe, Germany. The culture media were sterilized by autoclaving (LABOKLAV 25, SHP Steriltechnik, Magdeburg, Germany). Liquid cultures of *Staphylococcus xylosus* DSM 6179 (Schleifer and Kloos 1975) and *Escherichia coli* DH5α were grown overnight (130 rpm, 37 °C) in a MaxQ^TM^ 4000 orbital shaker (ThermoScientific, USA). Then, log-phase liquid cultures of bacteria were prepared by inoculating (5% *V*/*V*) sterile TSB and LB culture media from the overnight cultures of *S*. *xylosus* and *E*. *coli*, respectively. Bacteria were grown under standard conditions (180 rpm, 37 °C) until the fresh cultures reached an optical density of 0.6 at 600 nm (cell density meter WPA Biowave, Cambridge, UK), which indicated the logarithmic growth phase of the bacterial cultures.

MIC experiments were performed in 96-well microplates (Sarstedt, Hildesheim, Germany). First, 190 µL of silver nanoparticle-containing culture medium per well was mixed with 10 µL of the appropriate bacterial log-phase culture. The plate was then incubated overnight with gentle rotation in an orbital shaker (90 rpm, 37 °C). The MIC values were determined spectrophotometrically after 24 h of incubation (HiPo MPP-96 microplate reader; Biosan, Riga, Latvia) at 620 nm. Each sample was prepared and measured in triplicates. As a reference, AgNO_3_ dissolved in culture media was investigated. The silver concentrations used were 0, 5, 10, 15, 25, 50, 75, and 100 µg mL^−1^. Parallel to the MIC experiments, CFU (colony forming unit) values of the log-phase cultures were determined on agar plates after an overnight incubation at 37 °C (Heratherm^TM^ Compact, ThermoScientific, USA) to determine culture viability and bacterial cell dose per well. Bacterial colonies were counted with an SC6+ digital colony counter (Stuart, London, UK).

## 3. Results

**Figure 1:**
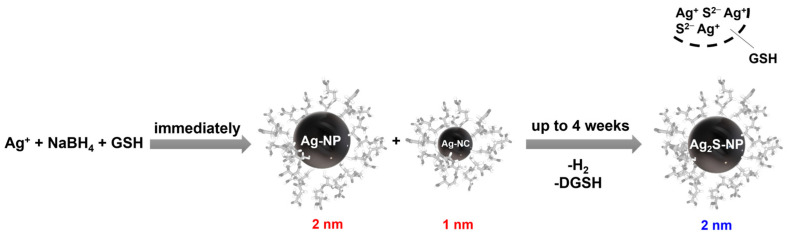
Proposed reaction scheme for the formation of ultrasmall silver sulfide nanoparticles via a mixture of silver nanoparticles (AgNP) and silver nanoclusters (AgNC). Over time, GSH eliminates H_2_S and is released into the solution as dehydrosulfoglutathione (DGSH).

A mixture of ultrasmall silver nanoparticles and silver nanoclusters is formed by the reduction of silver nitrate with NaBH_4_ in the presence of glutathione and then converted over several weeks into ultrasmall silver sulfide nanoparticles, as outlined in Figure 1. This reaction mechanism is discussed in the following sections and is supported by experimental evidence. In the following sections, we denote the silver nanoparticles with a maximum age of a few days as “freshly prepared nanoparticles” and the silver nanoparticles after complete conversion (at least 4 weeks dispersion in water at 4 °C) as “aged nanoparticles”.

**Figure 2:**
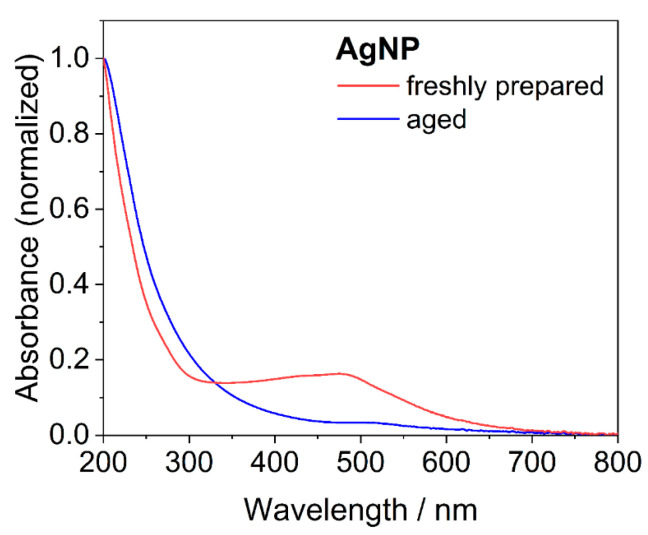
UV-Vis spectra of freshly prepared and aged silver nanoparticles (AgNP). Freshly prepared ultrasmall nanoparticles showed an absorbance at 480 nm, which is characteristic of silver nanoclusters.

**Figure 3:**
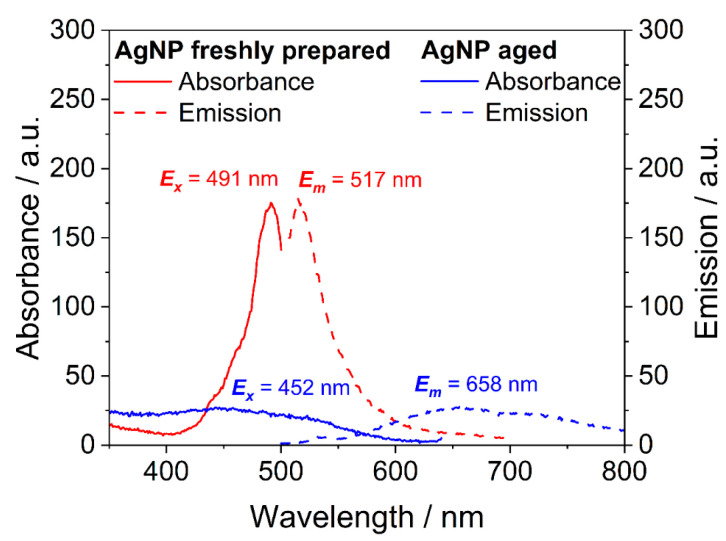
Fluorescence spectra of freshly prepared and aged silver nanoparticles (AgNP). The freshly prepared sample contained silver nanoclusters that caused a distinct absorption and fluorescence emission in the visible range. In contrast, the absorbance and emission of aged nanoparticles were both weak, indicating that the silver nanoclusters disappeared.

After synthesis, the sample of ultrasmall silver nanoparticles contained silver nanoclusters, which was obvious from the distinct red color of the dispersion. This was supported by UV (Figure 2) and fluorescence spectroscopy (Figure 3). Freshly prepared nanoparticles showed an absorption band at 480 nm that is characteristic of silver clusters (see below). Excitation at 491 nm led to a strong fluorescence with an emission maximum of 517 nm. In contrast, aged nanoparticles did not absorb light in this range and also did not give a significant fluorescence. Over time (weeks) in dispersion, the fluorescence progressively vanished, i.e., the absorbing and fluorescing silver nanoclusters disappeared.

The nature of the silver nanoclusters is difficult to determine from optical spectroscopy only. Absorption and emission wavelengths of metal nanoclusters depend on the cluster size and the capping ligands [38]. By controlling the size of the nanoclusters and the ligand-to-metal ratio during synthesis, photoemission can be varied from the UV region to the NIR region [39]. It was shown that even a difference of one silver atom in a silver nanocluster led to a shift in the emission color from blue to red [40]. DNA-stabilized silver nanoclusters changed their emission wavelength when the sequence of the DNA was changed [41]. Temperature-dependent shifts and intensity changes in the emission bands were also reported [42].

Silver nanoclusters are often prepared with glutathione as a capping ligand and sodium borohydride as a reducing agent [43,44,45]. Usually, they consist of a mixture of different cluster sizes, which can be separated by reversed-phase high-performance liquid chromatography (RP-HPLC). Ramsay et al. isolated three different silver nanocluster species from a reaction mixture with this method [45]. Each exhibited distinct optical properties by UV-Vis and fluorescence spectroscopy. They were assigned to the stoichiometries Ag_15_GSH_11_, Ag_32_GSH_19_, and a mixture of both by mass spectroscopy. Ag_15_GSH_11_ showed similar absorption and emission spectra, as shown in Figure 3, with an absorption maximum at 445 nm and an emission maximum at 680 nm [45]. Ag_32_GSH_19_ has an absorption maximum at about 485 nm [46,47,48,49]. Interestingly, the Stokes shift reported for Ag-GSH clusters was higher than in our case (25 nm; Figure 3). Ashenfelter et al. reported an emission at about 650 nm for Ag_11_GSH_7_, Ag_15_GSH_11_, and Ag_32_GSH_19_ after excitation with 420 nm [47]. They also showed that the emission originates from the metal–thiolate interface and not from the metal core [47].

In summary, we can conclude that the optical properties of freshly prepared silver nanoparticles are due to silver nanoclusters that disappear during aging. It is very likely that a mixture of silver nanoclusters is present. If we tentatively assume a cluster size between Ag_15_ and Ag_32_ (as these are particularly stable silver clusters [48]), this corresponds to a particle size of about 1 nm.

**Figure 4:**
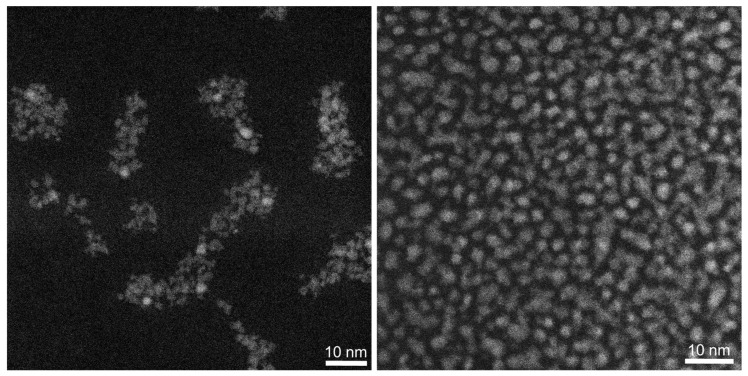
HAADF-STEM images of freshly prepared (**left**) and of aged silver nanoparticles (**right**).

High-angle annular dark-field scanning transmission electron microscopy (HAADF-STEM) was applied to elucidate the size of the silver nanoclusters. It showed that freshly prepared and aged silver nanoparticles were very similar in size and shape with average diameters of 2.2 ± 0.5 nm (freshly prepared) and 2.3 ± 0.7 nm (aged particles) (Figure 4). Thus, the mostly globular particles did not change significantly in size during prolonged dispersion in water. Silver nanoclusters were not found in the freshly prepared sample, in line with earlier observations of Ag_25_GSH_18_ nanoclusters that were completely unstable under the electron beam [50]. It is also conceivable that the formed silver sulfide nanoparticles may be reduced in the electron beam or at least undergo changes in crystallinity and composition [29].

**Figure 5:**
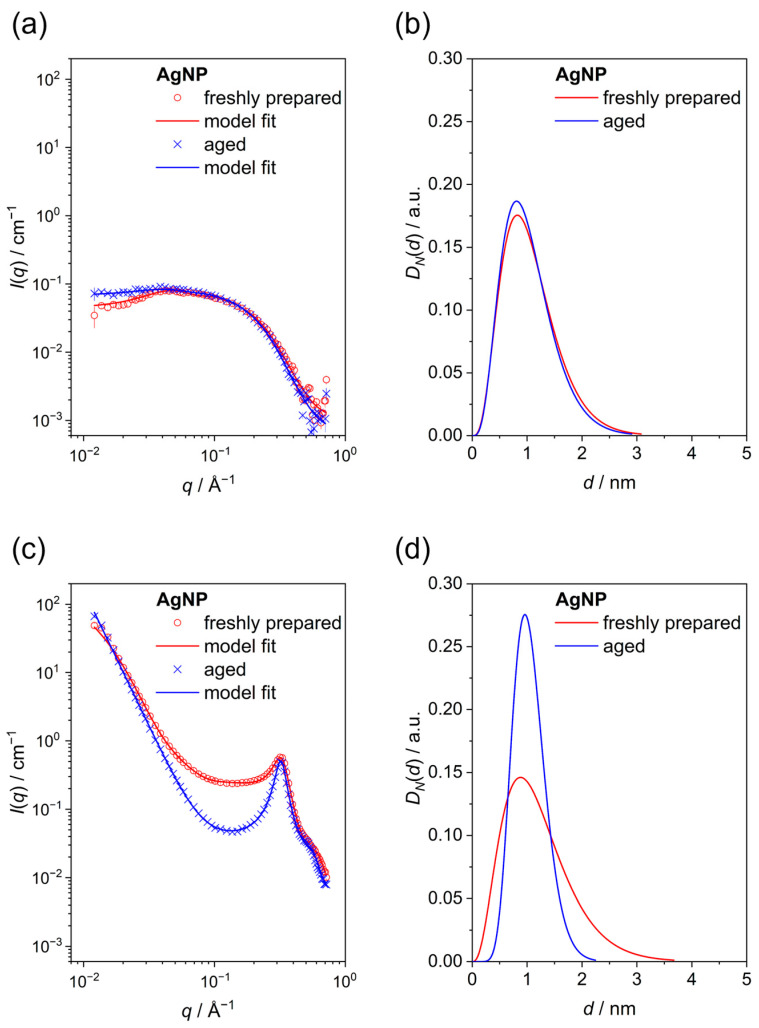
Small-angle X-ray scattering (SAXS) of freshly prepared and of aged silver nanoparticles. (**a**,**b**) show data of water-dispersed particles. (**c**,**d**) show data of freeze-dried particles (powders). (**a**,**c**) show primary scattering data (symbols) and model fits (lines). (**b**,**d**) show the computed particle size distributions.

Small-angle X-ray scattering is another method to probe the size distribution of dispersed particles. Lyophilized powders and water-dispersed particles were investigated (Figure 5). In all cases, particles with a core diameter of ~1 nm were detected. Interestingly, the aged sample had a tendency to smaller polydispersity, especially in the powder form. For the particles in dispersion, the effect of a repulsion structure factor, which decreases the scattering intensity at low angles, was included. By modeling the SAXS data with the hard spheres model, we obtained a hard sphere radius of 6.4 ± 0.6 nm and a volume fraction of 0.03 ± 0.01 for the freshly prepared sample. For the aged sample, 6.0 ± 0.2 nm and 0.073 ± 0.008 were obtained. Thus, a stronger structure factor effect was found for the aged sample.

For the powder samples, packing of the spherical particles led to a correlation peak around *q* ~ 0.32 Å^−1^. The correlation peak was modeled well by the hard spheres model, leading to a hard sphere radius of 1.0 ± 0.2 nm and a volume fraction of 0.399 ± 0.002 for the freshly prepared sample and 1.0 ± 0.2 nm and 0.455 ± 0.002 for the aged sample. Therefore, similarly to the nanoparticles in dispersion, the aged samples had stronger interactions that led to a better packing of the spherical particles. These correlation peaks can be considered as Bragg diffraction peaks. The application of Bragg’s law gave an interplanar distance of ~2.0 nm, which is twice the hard sphere radius. This indicates that the spheres were very well packed in the powder.

Finally, the low-angle region of the SAXS data of the powder samples was well-described by a fractal model. In both cases, we obtained a fractal dimension *D*_FR_ of 3.0, indicating a volume fractal. However, for the freshly prepared sample, we obtained an overall fractal size *ζ* of ~7 nm and a scale factor for the fractal contribution *SC_FR_* of 0.002. The values for the aged sample were ~88 nm and 0.4. This indicates the formation of bigger aggregates with larger fractions for the aged sample.

**Figure 6:**
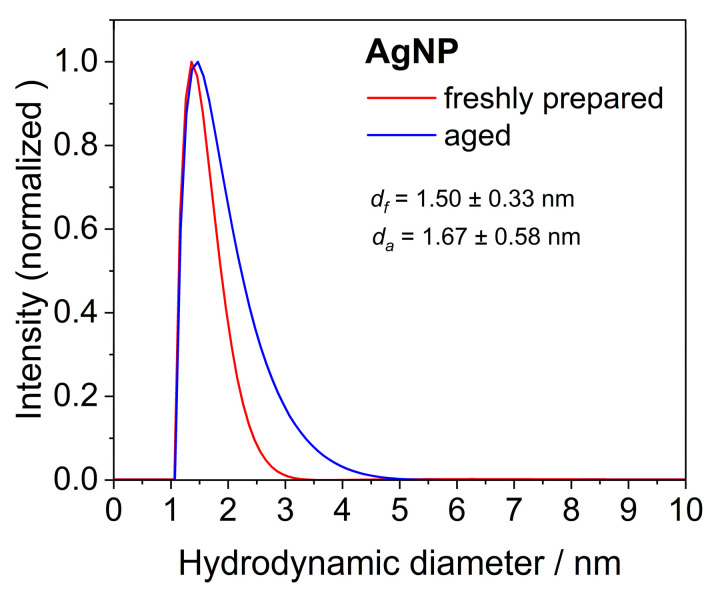
Differential centrifugal sedimentation (DCS) of freshly prepared and aged silver nanoparticles, dispersed in water.

Differential centrifugal sedimentation (DCS) also was not able discriminate between silver nanoparticles and silver nanoclusters in the freshly prepared samples, obviously as their sizes were too similar. However, the increase in particle size in aged nanoparticles indicated an increasing proportion of larger nanoparticles (Figure 6).

We conclude that the freshly prepared sample consists of a mixture of ultrasmall nanoparticles with a size of about 2 nm and an unknown but significant fraction of optically active silver nanoclusters, which can only be spectroscopically detected. Table 1 summarizes all particle size distribution data of freshly prepared and aged silver nanoparticles.

Upon storage in dispersion, the silver nanoclusters disappeared because their color and fluorescence vanished. It is not clear whether they dissolved by an Ostwald ripening process or whether they agglomerated with larger silver nanoparticles. It has already been reported that it is challenging to synthesize stable water-dispersed silver nanoclusters because they tend to aggregate [51]. It has also been demonstrated that glutathione-capped silver nanoclusters tend to grow over time, with Ag_15_GSH_11_ and Ag_32_GSH_19_ being particularly stable [48]. This was also found in a comprehensive study on the stability of glutathione-capped silver nanoclusters, based on optical spectroscopy and mass spectroscopy [46]. In situ small-angle X-ray scattering (SAXS) showed that larger polyacrylic acid-stabilized silver nanoparticles (3.2 nm) were etched by glutathione via very small particles (or complexes) that grew to larger particles (60 to 70 silver atoms; 1.3 nm) over time [52]. The microscopic mechanisms that are behind these conversions are still unclear, but these results confirm that there are considerable dynamics in such a mixture of nanoclusters and nanoparticles.

**Figure 7:**
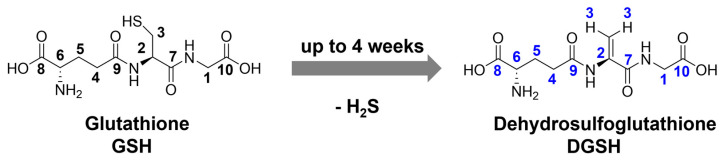
Elimination of H_2_S from glutathione by the conversion of the central cysteine into dehydroalanine, resulting in dehydrosulfoglutathione (DGSH). DGSH leaves the nanoparticle surface because the binding sulfur atom is lost. The black numbers denote carbon atoms and corresponding protons in glutathione; the blue numbers denote carbon atoms and corresponding protons in dehydrosulfoglutathione as indicated in the NMR spectra.

The second process that occurred in the dispersed nanoparticles was caused by the capping ligand glutathione. Glutathione can eliminate hydrogen sulfide (H_2_S) during immersion in water (Figure 7). The elimination of H_2_S from cysteine to dehydroalanine is well-known [53,54] and also possible for glutathione [55]. For the sake of brevity, we denote glutathione after H_2_S elimination in the following sections as “dehydrosulfoglutathione” and abbreviate it as DGSH (rational name: γ-glutamyldehydroalanylglycine). Dehydroalanine is a non-natural dehydroamino acid that has a C=C double bond. The tripeptide glutathione transfers its sulfur anchor atom to silver by H_2_S elimination and leaves the nanoparticle as a dissolved DGSH molecule. However, the number of glutathione ligands that is still present efficiently prevents agglomeration or precipitation of the nanoparticles. This process is completed after four weeks of storage in aqueous dispersion at 4 °C. In the following sections, we demonstrate how this mechanism was elucidated.

**Figure 8:**
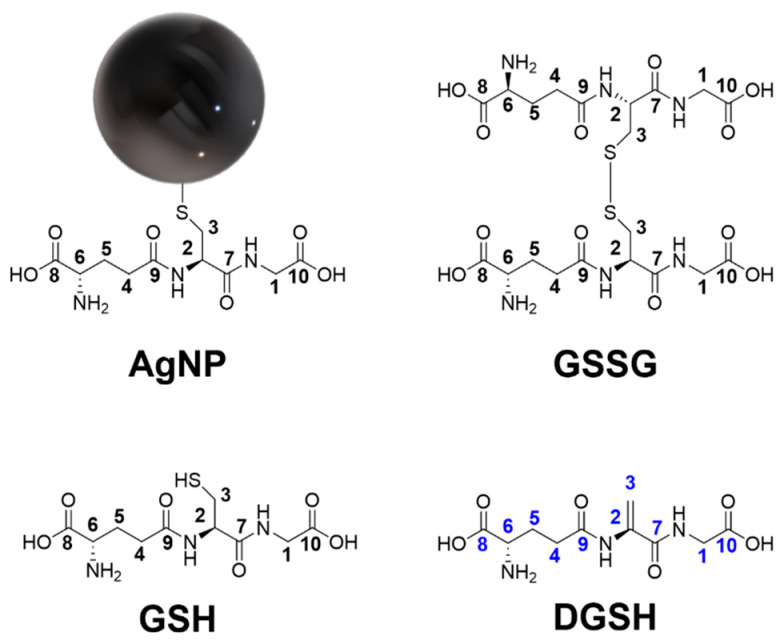
Different species in the ligand shell of silver nanoparticles. Initially, the surface of the silver nanoparticles (AgNP) is coated with glutathione (GSH) that binds via the central cysteine. Over time, cysteine eliminates H_2_S, and the tripeptide dehydrosulfoglutathione (DGSH) is released into the solution. Glutathione-disulfide (GSSG) is an oxidation product of glutathione. Carbon atoms and attached hydrogen atoms are labeled as assigned in the NMR spectra, e.g., H1 protons are attached to the C1 carbon atom. The black numbers denote carbon atoms and corresponding protons in glutathione and GSSG; the blue numbers denote carbon atoms and corresponding protons in dehydrosulfoglutathione as indicated in the NMR spectra.

**Figure 9:**
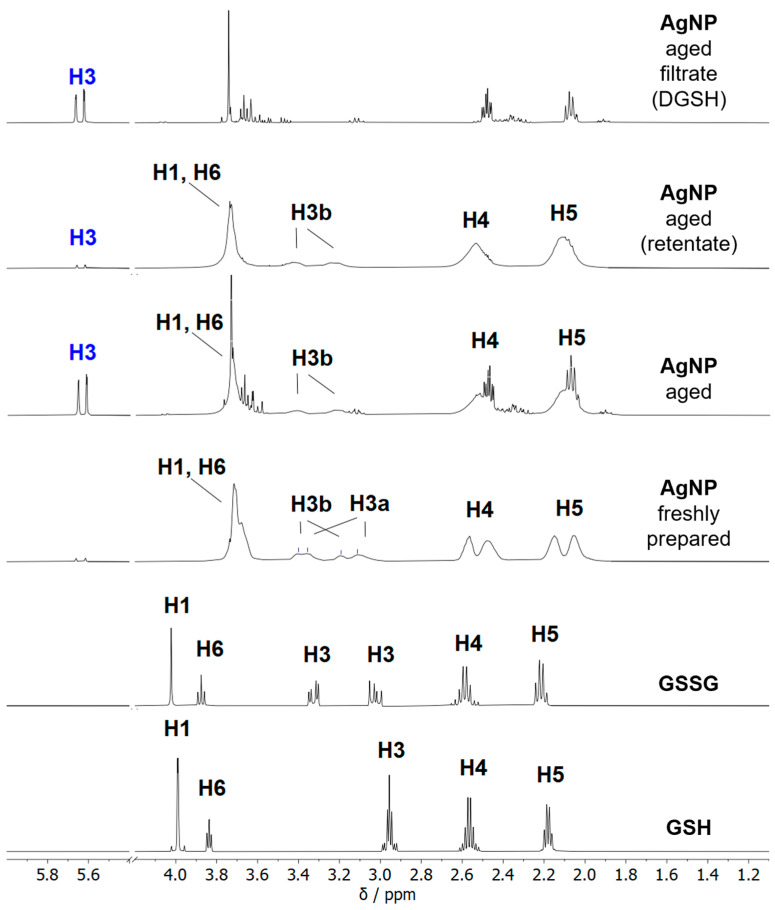
The ^1^H NMR spectra of freshly prepared and aged silver nanoparticles (400 MHz, 90% H_2_O, 10% D_2_O, pH 8.5) as well as of the ligand molecules GSH and GSSG. Soluble species were separated from the aged nanoparticles by spin filtration. The aged nanoparticles in the retentate were still coated by GSH, as indicated by the broad peaks. In contrast, the filtrate from aged nanoparticles showed dissolved dehydrosulfoglutathione that was formed by the elimination of H_2_S from the central cysteine in glutathione (note the H3 protons of DGSH colored in blue). The range between 4.2 and 5.4 ppm was cut off in all spectra as no peaks were present in this region.

NMR spectroscopy allows for probing the ligand shell of ultrasmall silver nanoparticles with high accuracy [16]. All relevant species are shown in Figure 8. Figure 9 shows the ^1^H-NMR spectra of dissolved GSH, dissolved glutathione-disulfide (GSSG; oxidized GSH connected via a disulfide bridge), dispersed freshly prepared and aged silver nanoparticles, and the filtrate and retentate of aged nanoparticles. Care was taken to perform all NMR experiments at the same pH (8.5) as the NMR peaks shift with pH. The assignment of the peaks of GSH in solution and on nanoparticles was consistent with earlier results [16,17]. The spectrum of freshly prepared nanoparticles did not show any sharp signals, confirming the absence of dissolved GSH. 

Distinct changes occurred in the NMR spectra of silver nanoparticles during several weeks of dispersion. After four weeks at 4 °C, no further changes were detected (see also Figure S1). Therefore, all silver particles above this age can be considered aged particles. The signal of the H3 protons (β-H) was split because of the chirality of the glutathione. The signal splitting of the β-H protons was also reported by Wu et al. for the Au_25_GSH_18_ nanocluster [56,57] and by Udayabhaskararao et al. for the Ag_32_GSH_19_ nanocluster [43]. Surprisingly, two sets of H3 protons, denoted as H3a and H3b, were found in the freshly prepared nanoparticles. First, the H3a signals at 3.35 and 3.11 ppm disappeared, whereas the H3b signals at 3.40 and 3.19 ppm were still present. The disappearing H3a proton signals were similar to those reported for Ag_32_GSH nanoclusters, suggesting that they belonged to the silver nanoclusters [43].

Two signals were observed for H4 (2.62 ppm, 2.55 ppm) and H5 protons (2.23 ppm, 2.14 ppm). This is due to the presence of silver nanoparticles and silver nanoclusters in the freshly prepared sample. For the COSY and HSQC spectra, no definite statement can be made as to whether the protons are located in the same cluster or two different particles because of the strong broadening of the signals and the proximity of the signals. However, because the signal losses also occurred at 2.14 and 2.55 ppm during storage (Figure S1), there is a high probability that these can be assigned to the silver nanoclusters that vanish over time. The signals at 2.14 and 2.55 ppm also correspond to results reported for the Ag_32_GSH nanocluster [43]. Furthermore, the occurrence of sharp signals in the spectrum of aged nanoparticles indicates that the ligand was released from the nanoparticle surface into the solution. A comparison with the spectra of dissolved GSH and GSSG showed that the dissolved species was neither GSH nor GSSG. Thus, an elimination of GSSG from the nanoparticle surface can be excluded. Notably, the doublet at 5.62/5.67 ppm was particularly prominent and indicated alkene protons.

Aged silver nanoparticles were separated by spin filtration from all dissolved species. The retentate contained the glutathione-coated silver nanoparticles as expected. The filtrate contained dissolved dehydrosulfoglutathione (DGSH), as confirmed in the ^13^C and 2D-NMR spectroscopy.

**Figure 10:**
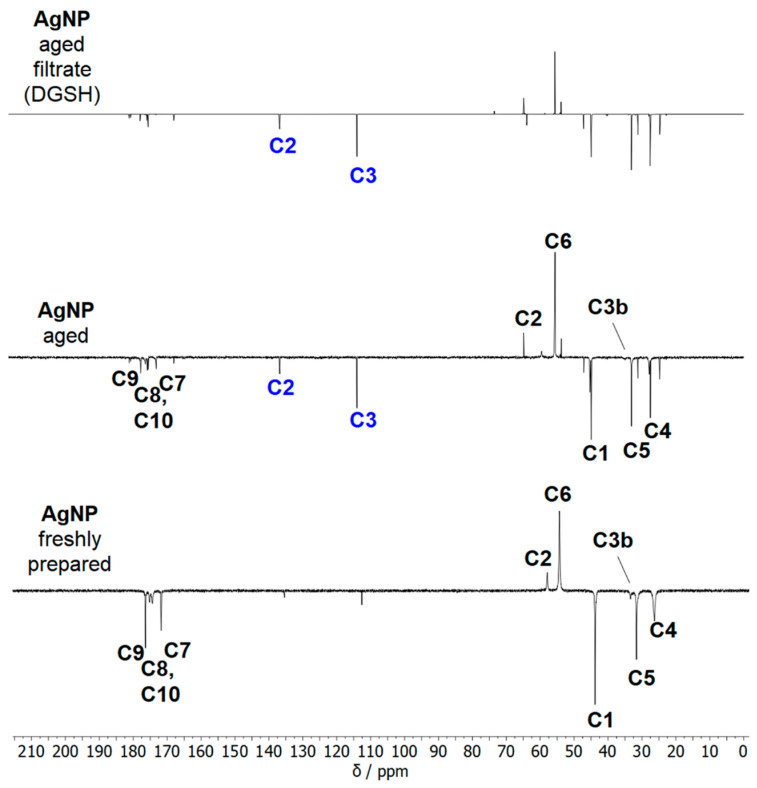
The ^13^C NMR DEPT Q spectra of freshly prepared and aged silver nanoparticles, as well as of the filtrate of aged nanoparticles (600 MHz, 90% H_2_O, 10% D_2_O, pH 8.5). The C2 and C3 signals labeled in blue belong to dissolved DGSH.

The ^13^C-NMR-DEPT Q spectra of freshly prepared and aged silver nanoparticles, as well as of the filtrate of aged nanoparticles, are shown in Figure 10. New ^13^C-signals at 112.6 ppm and 135.35 ppm appeared in aged nanoparticles and were also found in the filtrate. These chemical shifts are typical for C=C groups. Thus, the filtrate of aged nanoparticles contains mostly dissolved DGSH that detached from the nanoparticles.

**Figure 11:**
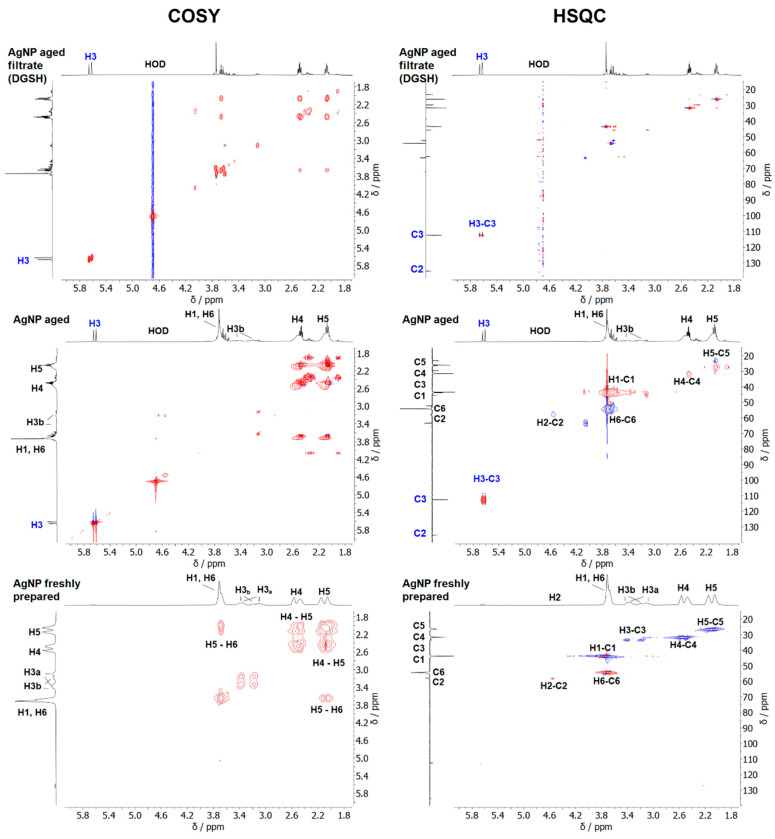
The ^1^H-^1^H COSY NMR spectra and ^1^H-^13^C HSQC NMR spectra of freshly prepared silver nanoparticles, aged silver nanoparticles, and the filtrate of aged nanoparticles. The filtrate consists mostly of dissolved DGSH (600 MHz, 90% H_2_O, 10% D_2_O; pH 8.5). Red colours denote CH_2_ groups. Blue colours denote CH groups.

The 2D-NMR spectra of the different compounds are shown in Figure 11. The H3 protons assigned to DGSH had a ^1^J coupling in the HSQC spectra with the ^13^C signal at 112.6 ppm. The phase in the HSQC spectrum indicates that this carbon atom belongs to a CH_2_ group (see the H4/H5 phase color), and the ^13^C-DEPT Q spectrum indicates that the carbon atom at 135.42 ppm is quaternary or a CH_2_-group. The ^2^J-coupling is visible in the HMBC spectrum (Figure S2) with the C2 signal at 133.06 ppm and a carbonyl C atom at 169.48 ppm. This carbon signal can be assigned to the carbonyl carbon at position C7 of DGSH. Thus, the presence of dissolved dehydrosulfoglutathione in aged nanoparticles is confirmed.

GSH was still present on the surface of aged nanoparticles, which are colloidally stable, as demonstrated by SAXS and DCS. Each silver nanoparticle (2 nm) contains about 250 silver atoms [58] and carries about 171 GSH ligands, as shown earlier by a combination of atomic absorption spectroscopy and NMR spectroscopy [16].

The ratio of GSH to DGSH in the aged nanoparticles was estimated by NMR spectroscopy by integrating the ^1^H NMR spectrum of aged nanoparticles (Figure 9). This was performed as follows: If we set the DGSH concentration to *x*, the concentration of the remaining GSH is (1 − *x*). The integral of H3 protons for DGSH (5.5 to 5.8 ppm) corresponds to 2*x* protons. The integral of the combined H5 protons (1.9 to 2.2 ppm) corresponds to 2*x* protons from DGSH and 2·(1 − *x*) protons from GSH, i.e., in total, two protons. Thus, the ratio of the integrals H3 (DGSH) to H5 (DGSH + GSH) equals 2*x*/2 = *x*. Experimentally, this integral ratio was 1:13.97, i.e., the percentage of DGSH was about 1/(1 + 13.97) = 7%. Consequently, about 12 out of 171 GSH ligands were released as DGSH into solution and about 159 remained on the nanoparticle surface. 

The elimination of H_2_S from cysteine leads to the formation of silver sulfide Ag_2_S. If 12 GSH ligands are released, as estimated above, they produce 12 H_2_S molecules that can oxidize 24 silver atoms to silver sulfide. This is about 10% of the total number of silver atoms (250) of each nanoparticle. It is reasonable to assume that these are silver atoms on the nanoparticle surface. Tentatively, we can formulate the overall reaction to

Ag_250_GSH_171_ ➝ Ag_250_S_24_GSH_159_ + 12 H_2_ + 12 DGSH

**Figure 12:**
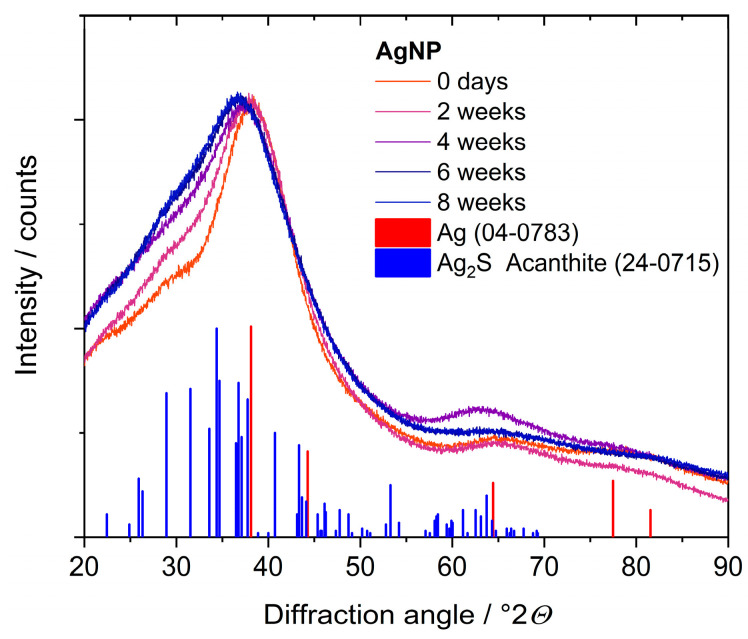
X-ray powder diffraction of silver nanoparticles, taken after different immersion times in water for up to 8 weeks. The main peak at 37°2*θ* is the (111) peak of silver (fcc). The increasing shoulder of this peak towards lower angles indicates the presence of crystalline Ag_2_S (acanthite).

Next, we consider the core of the nanoparticle in detail. X-ray powder diffraction showed very broad peaks because of the small particle size that was close to the expected peak for metallic silver. Upon aging in dispersion, a shoulder on the left side of the main diffraction peak indicated the formation of crystalline Ag_2_S (acanthite). Clearly, X-ray powder diffraction reached its limits with very small and disordered particles (Figure 12).

**Figure 13:**
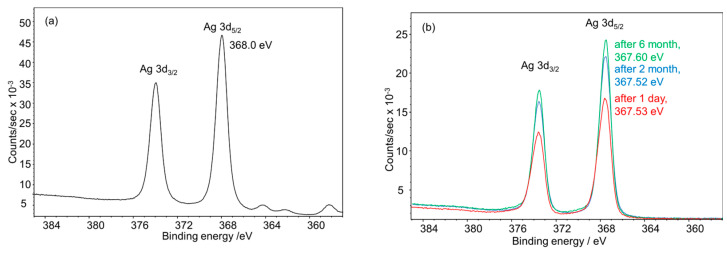
High-resolution X-ray photoelectron Ag 3d spectra collected for (**a**) macroscopic Ag_2_S for comparison [59] and (**b**) freshly prepared and aged silver nanoparticles. The spectrum of Ag_2_S was collected with a non-monochromatic Al X-ray source, explaining the satellite peaks at lower binding energies.

The assignment of different oxidation states for silver by X-ray photoelectron spectroscopy (XPS) is very challenging as the binding energies of the different species for Ag^0^, Ag_2_O, and Ag_2_S differ only slightly. Compared with the spectra of bulk Ag_2_S (measured with a non-monochromatic Al dual anode [59]), the spectra of all nanoparticles measured in this work (monochromatic Al anode) were significantly broadened, and the peak shape was more asymmetric. The binding energies of the main Ag 3d_5/2_ signals of the Ag nanoparticles measured before and after aging were lower than expected for metallic silver at 368.3 eV [60]. The data confirm that silver was quantitatively oxidized to silver sulfide, i.e., Ag_2_S with Ag(+I), in the aged nanoparticles (Figure 13). Remarkably, the freshly prepared nanoparticles also consisted of Ag(+I), as reported earlier [59], matching very well with the control sample, i.e., macroscopic Ag_2_S. However, as the photopeaks are much broader and more asymmetric in shape, a contribution of metallic silver at the high binding energy side of the Ag 3d peaks cannot be excluded, but it is minor in any case. We ascribe the high degree of oxidation of silver in the silver nanoparticles to the presence of formally oxidized silver atoms on the nanoparticle surface that are bound to GSH (note the ratio of Ag:GSH of 250:171). Notably, changes in the ultrasmall particles and nanoclusters under XPS conditions (X-ray irradiation, ultrahigh vacuum) might also occur.

The conversion of water-dispersed Ag_25_GSH_18_ silver nanoclusters into Ag_2_S nanoparticles upon heating was reported by Remya et al. back in 2012 [50]. The time scale for complete conversion was about 30 h at 80 °C. They postulated a release of the ligand by breaking the carbon–sulfur bond with an unclear mechanism, based on mass spectrometric results (fragmentation of glutathione). However, the elimination of H_2_S to dehydrosulfoglutathione we report here perfectly matches their observations, which were mainly based on UV spectroscopy and mass spectrometry. They showed by TEM that the formed silver sulfide nanoparticles had a diameter of about 3 nm with good crystallinity (acanthite polymorph) [50].

**Figure 14:**
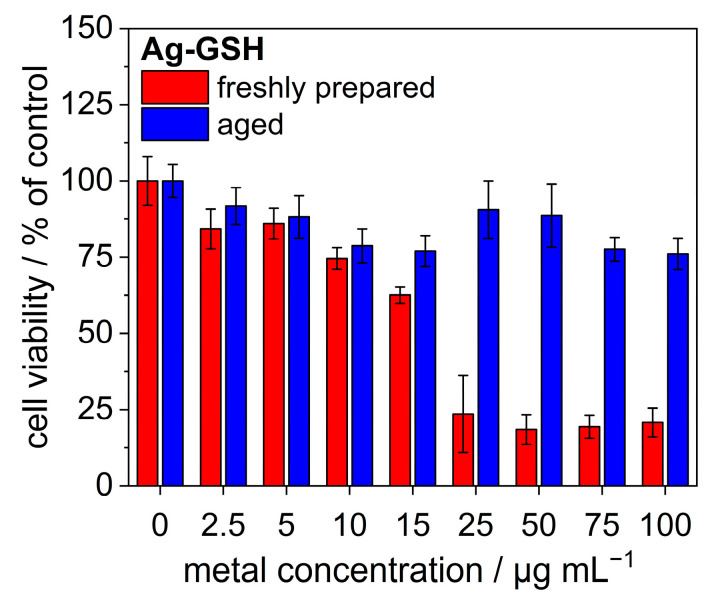
Cytotoxicity of freshly prepared and aged silver nanoparticles assessed by an MTT test on HeLa cells.

Finally, the presence of silver nanoclusters considerably increased the cytotoxicity of the nanoparticles. Figure 14 shows data on the cytotoxicity of freshly prepared and aged silver nanoparticles. Obviously, aging decreased the cytotoxicity. This can be related to surface passivation by the formation of a sulfide layer, as reported earlier for larger silver nanoparticles. Such a conversion can occur in the presence of sulfide, e.g., under environmental conditions where sulfide frequently occurs [61,62]. In these cases, sulfide is an external agent, whereas, in the mechanism we found here, it is formed by dehydrosulfuridation of the capping ligand, i.e., from an intrinsic source.

The antibacterial effect of the nanoparticles was assessed on the model bacterial species *Escherichia coli* and *Staphylococcus xylosus*, which differ in their cell envelope structure. We found MIC values for *E. coli* of 6 to 10 µg mL^−1^ for AgNO_3_ and freshly prepared silver nanoparticles_._ For *S. xylosus*, freshly prepared nanoparticles were even more toxic (1 to 5 µg mL^−1^) than pure AgNO_3_ (15 to 25 µg mL^−1^). For both bacterial strains, aged silver nanoparticles were not toxic up to a concentration of 100 µg mL^−1^ (Table 2). In conclusion, the freshly prepared nanoparticles were much more bactericidal than the aged ones in accordance with the effect on eukaryotic cells. This can only be due to the presence of the silver nanoclusters in the freshly prepared sample. Silver nanoclusters have shown considerable activity towards *E. coli* (10 to 15 µg mL^−1^ [14]). In contrast, aged ultrasmall silver nanoparticles are much less cytotoxic as they do not release silver ions [37].

Thus, the aged particles mostly consisted of silver sulfide, which is a well-known material in materials science, mostly studied for its semiconducting properties in combination with low water solubility. The structure and syntheses of (usually larger) silver sulfide nanoparticles, including their semiconducting properties, were comprehensively discussed by Sadovnikov and Gusev [63]. In general, different synthetic pathways have been explored (usually a synthesis from sulfide ions and silver ions) [64,65,66], also leading to atom-sharp clusters [67], and they have also been described theoretically [68,69]. Because of their luminescent properties and their low cytotoxicity, silver nanoparticles have been discussed for imaging in biomedicine [64,70,71]. Their high X-ray density also permits X-ray imaging of the body by computer tomography [72].

Aging was completed after four weeks of dispersion in water at 4 °C. Although we did not perform a kinetic study, it is likely that the process occurs much faster at room temperature.

## 4. Conclusions

Ultrasmall silver nanoparticles stabilized by the cysteine-containing ligand glutathione undergo considerable changes on the timescale of weeks during immersion in water. Immediately after synthesis, they contain a fraction of smaller silver nanoclusters as detectable by their color (light absorption) and fluorescence. These disappear over time, either by dissolution or by agglomeration with larger silver nanoparticles. Cysteine from the capping ligand glutathione eliminates hydrogen sulfide that reacts with metallic silver to silver sulfide. The modified ligand dehydrosulfoglutathione leaves the surface of the nanoparticle as it has lost its anchoring sulfur atom and goes into the solution. These changes cannot be elucidated by size-selective methods like disc centrifugal sedimentation, small-angle X-ray scattering, or transmission electron microscopy as silver nanoclusters and silver nanoparticles cannot be separately detected. However, these processes can be quantitatively followed by NMR spectroscopy in dispersion. The presence of silver nanoclusters leads to an increased cytotoxicity towards cells and bacteria in comparison with aged nanoparticles.

Cysteine-containing capping ligands like glutathione are often used to stabilize metal nanoparticles. Thus, the presented elimination mechanism probably occurs frequently in samples of noble metals and remains undetected because only NMR spectroscopy can unequivocally elucidate it. However, NMR spectroscopy is rarely used to characterize ultrasmall nanoparticles and is not applicable to larger nanoparticles. This selective oxidation of metals by glutathione to a metal sulfide is more likely to occur for less noble metals like silver because they can be oxidized more easily than very noble metals like gold or platinum. In terms of stoichiometry, this effect will be more significant for ultrasmall nanoparticles than for conventional (e.g., plasmonic) nanoparticles because of the higher percentage of atoms on the particle surface. In conclusion, it is likely that this elimination mechanism may be more common than usually assumed, especially for less noble metals. Therefore, studies where nanoparticles are prepared as biologically active agents (e.g., silver as a bactericidal agent) should take possible aging effects into account.

## Figures and Tables

**Table 1:** Summary of the particle size data obtained by different methods.

Nanoparticle Type	Silver Nanoparticles (Freshly Prepared)	Silver Nanoparticles (Aged)
Hydrodynamic diameter by DCS/nm	1.5 ± 0.3	1.7 ± 0.6
Core diameter by SAXS (dispersed particles)/nm	1.0 ± 0.4	1.2 ± 0.2
Core diameter by SAXS (powder)/nm	1.1 ± 0.3	1.1 ± 0.15
Core diameter by HAADF-STEM/nm	2.2 ± 0.5	2.3 ± 0.7

**Table 2:** MIC values determined for *E. coli* DH5α and *S. xylosus* DSM 6179 after 24 h of incubation with freshly prepared or aged silver nanoparticles (concentrations given in µg silver mL^−1^). *E*. *coli* (CFU: 1.1 × 10^8^ cells mL^−1^; cell dose: 1.1 × 10^6^ cells per well), *S*. *xylosus* (CFU: 5 × 10^7^ cells mL^−1^; cell dose: 5 × 10^5^ cells per well).

Sample	*E. coli* (Gram-Negative)	*S. xylosus* (Gram-Positive)
AgNO_3_	6 to 10	15 to 25
Freshly prepared silver nanoparticles	6 to 10	1 to 5
Aged silver nanoparticles	>100	>100

## Data Availability

The original contributions presented in this study are included in this article/Supplementary Materials, further inquiries can be directed to the corresponding authors.

## Supplementary Materials

The following supporting information can be downloaded at https://www.mdpi.com/article/10.3390/nano14171449/s1: Figure S1: Kinetic study by ^1^H NMR spectroscopy on ultrasmall silver nanoparticles dispersed in water at 4 °C. Figure S2: The ^1^H-^13^C HMBC NMR spectrum of aged silver nanoparticles.

## References

[1] Du X.S., Jin R.C. (2020). Atomic-precision engineering of metal nanoclusters. Dalton Trans..

[2] Yang J., Jin R.C. (2019). New advances in atomically precise silver nanoclusters. ACS Mater. Lett..

[3] Perala S.R.K., Kumar S. (2013). On the mechanism of metal nanoparticle synthesis in the Brust–Schiffrin method. Langmuir.

[4] Liz-Marzan L.M. (2013). Gold nanoparticle research before and after the Brust–Schiffrin method. Chem. Commun..

[5] Epple M., Rotello V.M., Dawson K. (2023). The why and how of ultrasmall nanoparticles. Acc. Chem. Res..

[6] Zarschler K., Rocks L., Licciardello N., Boselli L., Polo E., Garcia K.P., De Cola L., Stephan H., Dawson K.A. (2016). Ultrasmall inorganic nanoparticles: State-of-the-art and perspectives for biomedical applications. Nanomedicine.

[7] Wagner L.S., Prymak O., Schaller T., Beuck C., Loza K., Niemeyer F., Gumbiowski N., Kostka K., Bayer P., Heggen M. (2024). The molecular footprint of peptides on the surface of ultrasmall gold nanoparticles (2 nm) is governed by steric demand. J. Phys. Chem. B.

[8] Liu Z.H., Wu Z.N., Yao Q.F., Cao Y.T., Chai O.J.H., Xie J.P. (2021). Correlations between the fundamentals and applications of ultrasmall metal nanoclusters: Recent advances in catalysis and biomedical applications. Nano Today.

[9] Du Y., Sheng H., Astruc D., Zhu M. (2020). Atomically precise noble metal nanoclusters as efficient catalysts: A bridge between structure and properties. Chem. Rev..

[10] Rodrigues T.S., da Silva A.G.M., Camargo P.H.C. (2019). Nanocatalysis by noble metal nanoparticles: Controlled synthesis for the optimization and understanding of activities. J. Mater. Chem. A.

[11] Zhao J.B., Jin R.C. (2018). Heterogeneous catalysis by gold and gold-based bimetal nanoclusters. Nano Today.

[12] Draviana H.T., Fitriannisa I., Khafid M., Krisnawati D.I., Widodo, Lai C.H., Fan Y.J., Kuo T.R. (2023). Size and charge effects of metal nanoclusters on antibacterial mechanisms. J. Nanobiotechnol..

[13] Zheng K.Y., Yuan X., Goswami N., Zhang Q.B., Xie J.P. (2014). Recent advances in the synthesis, characterization, and biomedical applications of ultrasmall thiolated silver nanoclusters. RSC Adv..

[14] Jin J.C., Wu X.J., Xu J., Wang B.B., Jiang F.L., Liu Y. (2017). Ultrasmall silver nanoclusters: Highly efficient antibacterial activity and their mechanisms. Biomater. Sci..

[15] Lopez P., Lara H.H., Mullins S.M., Black D.M., Ramsower H.M., Alvarez M.M., Williams T.L., Lopez-Lozano X., Weissker H.C., Garcia A.P. (2018). Tetrahedral (T) closed-shell cluster of 29 silver atoms & 12 lipoate ligands, Ag_29_[R-alpha-LA)_12_]^(3-)^: Antibacterial and antifungal activity. ACS Appl. Nano Mater..

[16] Wolff N., Loza K., Heggen M., Schaller T., Niemeyer F., Bayer P., Beuck C., Oliveira C.L.P., Prymak O., Weidenthaler C. (2023). Ultrastructure and surface composition of glutathione-terminated ultrasmall silver, gold, platinum, and alloyed silver–platinum nanoparticles (2 nm). Inorg. Chem..

[17] Wetzel O., Prymak O., Loza K., Gumbiowski N., Heggen M., Bayer P., Beuck C., Weidenthaler C., Epple M. (2022). Water-based synthesis of ultrasmall nanoparticles of platinum group metal oxides (1.8 nm). Inorg. Chem..

[18] Wolff N., Beuck C., Schaller T., Epple M. (2024). Possibilities and limitations of solution-state NMR spectroscopy to analyze the ligand shell of ultrasmall metal nanoparticles. Nanoscale Adv..

[19] Marbella L.E., Millstone J.E. (2015). NMR techniques for noble metal nanoparticles. Chem. Mater..

[20] Salassa G., Burgi T. (2018). NMR spectroscopy: A potent tool for studying monolayer-protected metal nanoclusters. Nanoscale Horiz..

[21] Fan M., Han Y., Gao S.T., Yan H.Y., Cao L.Z., Li Z.H., Liang X.J., Zhang J.C. (2020). Ultrasmall gold nanoparticles in cancer diagnosis and therapy. Theranostics.

[22] Zeng C.J. (2018). Precision at the nanoscale: On the structure and property evolution of gold nanoclusters. Pure Appl. Chem..

[23] Jin R., Zeng C., Zhou M., Chen Y. (2016). Atomically precise colloidal metal nanoclusters and nanoparticles: Fundamentals and opportunities. Chem. Rev..

[24] Kim N.H., Hackett M.J., Park J., Hyeon T. (2014). Synthesis, characterization, and application of ultrasmall nanoparticles. Chem. Mater..

[25] Zhou M., Du X., Wang H., Jin R. (2021). The critical number of gold atoms for a metallic state nanocluster: Resolving a decades-long question. ACS Nano.

[26] Srinivasulu Y.G., Yao Q.F., Goswami N., Xie J.P. (2020). Interfacial engineering of gold nanoclusters for biomedical applications. Mater. Horiz..

[27] Sakthivel N.A., Dass A. (2018). Aromatic thiolate-protected series of gold nanomolecules and a contrary structural trend in size evolution. Acc. Chem. Res..

[28] Häkkinen H. (2012). The gold–sulfur interface at the nanoscale. Nat. Chem..

[29] Egerton R.F., Li P., Malac M. (2004). Radiation damage in the TEM and SEM. Micron.

[30] Vogt C., Meirer F., Monai M., Groeneveld E., Ferri D., van Santen R.A., Nachtegaal M., Unocic R.R., Frenkel A.I., Weckhuysen B.M. (2021). Dynamic restructuring of supported metal nanoparticles and its implications for structure insensitive catalysis. Nat. Commun..

[31] Zhang J., My Pham T.H., Gao Z., Li M., Ko Y., Lombardo L., Zhao W., Luo W., Züttel A. (2023). Electrochemical CO_2_ reduction over copper phthalocyanine derived catalysts with enhanced selectivity for multicarbon products. ACS Catal..

[32] Hammersley A.P. (2016). FIT2D: A multi-purpose data reduction, analysis and visualization program. J. Appl. Crystallogr..

[33] Oliveira C.L.P., Vorup-Jensen T., Andersen C.B.F., Andersen G.R., Pedersen J.S., Gomez M., Nogales A., Garcia-Gutierrez M.C., Ezquerra T.A. (2009). Discovering new features of protein complexes structures by small-angle X-ray scattering. Applications of Synchrotron Light to Scattering and Diffraction in Materials and Life Sciences.

[34] Teixeira J. (1988). Small-angle scattering by fractal systems. J. Appl. Crystallogr..

[35] Brust M., Fink J., Bethell D., Schiffrin D.J., Kiely C. (1995). Synthesis and reactions of functionalised gold nanoparticles. Chem. Commun..

[36] Brust M., Walker M., Bethell D., Schiffrin D.J., Whyman R. (1994). Synthesis of thiol-derivatised gold nanoparticles in a two-phase liquid-liquid system. Chem. Commun..

[37] Wolff N., Białas N., Loza K., Heggen M., Schaller T., Niemeyer F., Weidenthaler C., Beuck C., Bayer P., Prymak O. (2024). Increased cytotoxicity of bimetallic ultrasmall silver–platinum nanoparticles (2 nm) on cells and bacteria in comparison to silver nanoparticles of the same size. Materials.

[38] Yang T.Q., Peng B., Shan B.Q., Zong Y.X., Jiang J.G., Wu P., Zhang K. (2020). Origin of the photoluminescence of metal nanoclusters: From metal-centered emission to ligand-centered emission. Nanomaterials.

[39] Zheng J., Nicovich P.R., Dickson R.M. (2007). Highly fluorescent noble-metal quantum dots. Ann. Rev. Phys. Chem..

[40] Udaya Bhaskara Rao T., Pradeep T. (2010). Luminescent Ag_7_ and Ag_8_ clusters by interfacial synthesis. Angew. Chem. Int. Ed..

[41] Richards C.I., Choi S., Hsiang J.C., Antoku Y., Vosch T., Bongiorno A., Tzeng Y.L., Dickson R.M. (2008). Oligonucleotide-stabilized Ag nanocluster fluorophores. J. Am. Chem. Soc..

[42] Wang Z., Gupta R.K., Luo G.G., Sun D. (2020). Recent progress in inorganic anions templated silver nanoclusters: Synthesis, structures and properties. Chem. Record.

[43] Udayabhaskararao T., Bootharaju M.S., Pradeep T. (2013). Thiolate-protected Ag32 clusters: Mass spectral studies of composition and insights into the Ag–thiolate structure from NMR. Nanoscale.

[44] Bertorelle F., Hamouda R., Rayane D., Broyer M., Antoine R., Dugourd P., Gell L., Kulesza A., Mitric R., Bonacic-Koutecky V. (2013). Synthesis, characterization and optical properties of low nuclearity liganded silver clusters: Ag_31_(SG)_19_ and Ag_15_(SG)_11_. Nanoscale.

[45] Ramsay H., Simon D., Steele E., Hebert A., Oleschuk R.D., Stamplecoskie K.G. (2018). The power of fluorescence excitation–emission matrix (EEM) spectroscopy in the identification and characterization of complex mixtures of fluorescent silver clusters. RSC Adv..

[46] Desireddy A., Kumar S., Guo J.S., Bolan M.D., Griffith W.P., Bigioni T.P. (2013). Temporal stability of magic-number metal clusters: Beyond the shell closing model. Nanoscale.

[47] Ashenfelter B.A., Desireddy A., Yau S.H., Goodson T., Bigioni T.P. (2015). Fluorescence from molecular silver nanoparticles. J. Phys. Chem. C.

[48] Zaker Y., Ashenfelter B.A., Bhattarai B., Diemler N.A., Brewer T.R., Bigioni T.P. (2021). Sequential growth as a mechanism of silver-glutathione monolayer-protected cluster formation. Small.

[49] Zaker Y., Bhattarai B., Brewer T.R., Bigioni T.P. (2021). The role of oxidation during the synthesis of silver-glutathione monolayer-protected clusters. Small.

[50] Remya K.P., Udayabhaskararao T., Pradeep T. (2012). Low-Temperature thermal dissociation of Ag quantum clusters in solution and formation of monodisperse Ag_2_S nanoparticles. J. Phys. Chem. C.

[51] Xu H., Suslick K.S. (2010). Water-soluble fluorescent silver nanoclusters. Adv. Mater..

[52] Kästner C., Saloga P.E.J., Thünemann A.F. (2018). Kinetic monitoring of glutathione-induced silver nanoparticle disintegration. Nanoscale.

[53] Qiao Y., Yu G., Leeuwon S.Z., Liu W.R. (2021). Site-specific conversion of cysteine in a protein to dehydroalanine using 2-nitro-5-thiocyanatobenzoic acid. Molecules.

[54] Chalker J.M., Gunnoo S.B., Boutureira O., Gerstberger S.C., Fernandez-Gonzalez M., Bernardes G.J.L., Griffin L., Hailu H., Schofield C.J., Davis B.G. (2011). Methods for converting cysteine to dehydroalanine on peptides and proteins. Chem. Sci..

[55] Younis I.R., Elliott M., Peer C.J., Cooper A.J., Pinto J.T., Konat G.W., Kraszpulski M., Petros W.P., Callery P.S. (2008). Dehydroalanine analog of glutathione: An electrophilic busulfan metabolite that binds to human glutathione S-transferase A1-1. J. Pharmacol. Exp. Ther..

[56] Wu Z., Gayathri C., Gil R.R., Jin R. (2009). Probing the structure and charge state of glutathione-capped Au_25_(SG)_18_ clusters by NMR and mass spectrometry. J. Am. Chem. Soc..

[57] Wu Z., Jin R. (2009). Stability of the two Au−S binding modes in Au_25_(SG)_18_ nanoclusters probed by NMR and optical spectroscopy. ACS Nano.

[58] Mingos D.M.P. (2014). Gold Clusters, Colloids and Nanoparticles I.

[59] Wetzel O., Hosseini S., Loza K., Heggen M., Prymak O., Bayer P., Beuck C., Schaller T., Niemeyer F., Weidenthaler C. (2021). Metal–ligand interface and internal structure of ultrasmall silver nanoparticles (2 nm). J. Phys. Chem. B.

[60] Ferraria A.M., Carapeto A.P., do Rego A.M.B. (2012). X-ray photoelectron spectroscopy: Silver salts revisited. Vacuum.

[61] Levard C., Hotze E.M., Lowry G.V., Brown G.E. (2012). Environmental transformations of silver nanoparticles: Impact on stability and toxicity. Environ. Sci. Toxicol..

[62] Fletcher N.D., Lieb H.C., Mullaugh K.M. (2019). Stability of silver nanoparticle sulfidation products. Sci. Total Environ..

[63] Sadovnikov S.I., Gusev A.I. (2017). Recent progress in nanostructured silver sulfide: From synthesis and nonstoichiometry to properties. J. Mater. Chem. A.

[64] Ibrahim M., Camarero P., Ming L.Y., Haouari M., Amamou N., Haro-Gonzalez P., Hassen F. (2023). Wet chemical synthesis of TGA capped Ag_2_S nanoparticles and their use for fluorescence imaging and temperature sensing in living cells. RSC Adv..

[65] Lu F., Gong Y., Ju W.W., Cheng F., Zhang K.W., Wang Q., Wang W.J., Zhong J.B., Fan Q.L., Huang W. (2019). Facile one-pot synthesis of monodispersed NIR-II emissive silver sulfide quantum dots. Inorg. Chem. Commun..

[66] Manju C.K., Mohanty J.S., Sarkar D., Chennu S., Pradeep T. (2018). Towards atomically precise luminescent Ag_2_S clusters separable by thin layer chromatography. J. Mater. Chem. C.

[67] Bestgen S., Fuhr O., Breitung B., Chakravadhanula V.S.K., Guthausen G., Hennrich F., Yu W., Kappes M.M., Roesky P.W., Fenske D. (2017). [Ag_115_S_34_(SCH_2_C_6_H_4_ tBu)_47_(dpph)_6_]: Synthesis, crystal structure and NMR investigations of a soluble silver chalcogenide nanocluster. Chem. Sci..

[68] Tian Z.M., Song C.F., Wang C., Xu H.J., Guan Q.M. (2020). Structures and properties of [Ag(Ag_2_S)_n_]+clusters with n = 1–9: A density functional theory study. J. Nanoparticle Res..

[69] Song C.F., Tian Z.M. (2019). Systematic study on the structures and properties of (Ag_2_S)_n_ (n = 1–8) clusters. J. Mol. Model..

[70] Hsu J.C., Barragan D., Tward A.E., Hajfathalian M., Amirshaghaghi A., Mossburg K.J., Rosario-Berríos D.N., Bouché M., Andrianov A.K., Delikatny E.J. (2024). A biodegradable “one-for-all” nanoparticle for multimodality imaging and enhanced photothermal treatment of breast cancer. Adv. Healthc. Mater..

[71] Yang G., Wang Z.P., Du F.L., Jiang F.Y., Yuan X., Ying J.Y. (2023). Ultrasmall coinage metal nanoclusters as promising theranostic probes for biomedical applications. J. Am. Chem. Soc..

[72] Hsu J.C., Cruz E.D., Lau K.C., Bouché M., Kim J., Maidment A.D.A., Cormode D.P. (2019). Renally excretable and size-tunable silver sulfide nanoparticles for dual-energy mammography or computed tomography. Chem. Mater..

