# Supplementary material for: Conversion of Ultrasmall Glutathione-Coated Silver Nanoparticles during Dispersion in Water into Ultrasmall Silver Sulfide Nanoparticles
Source: Nanomaterials (Basel). 2024 Sep 5;14(17):1449. doi: 10.3390/nano14171449 (PMC11397201; doi:10.3390/nano14171449)
Supplement: Supplementary file 1 [file nanomaterials-14-01449-s001.zip › nanomaterials-3188723-supplementary.pdf]

# Supporting information

## Conversion of Ultrasmall Glutathione-Coated Silver Nanoparticles during Dispersion in Water into Ultrasmall Silver Sulfide Nanoparticles

Natalie Wolff 1, Oleg Prymak 1, Nataniel Białas 1, Torsten Schaller 2, Kateryna Loza 1, Felix Niemeyer 2, Marc Heggen 3, Claudia Weidenthaler 4, Cristiano L. P. Oliveira 5 and Matthias Epple [1,*]

[1] Inorganic Chemistry and Centre for Nanointegration Duisburg-Essen (CENIDE), University of Duisburg-Essen, Universitaetsstr. 5-7, 45117 Essen, Germany
[2] Organic Chemistry, University of Duisburg-Essen, Universitaetsstr. 5-7, 45117 Essen, Germany
[3] Ernst Ruska Centre for Microscopy and Spectroscopy with Electrons, Forschungszentrum Jülich, 52428 Jülich, Germany
[4] Max-Planck-Institut für Kohlenforschung, 45470 Mülheim an der Ruhr, Germany
[5] Institute of Physics, University of São Paulo, São Paulo 05508-090, Brazil

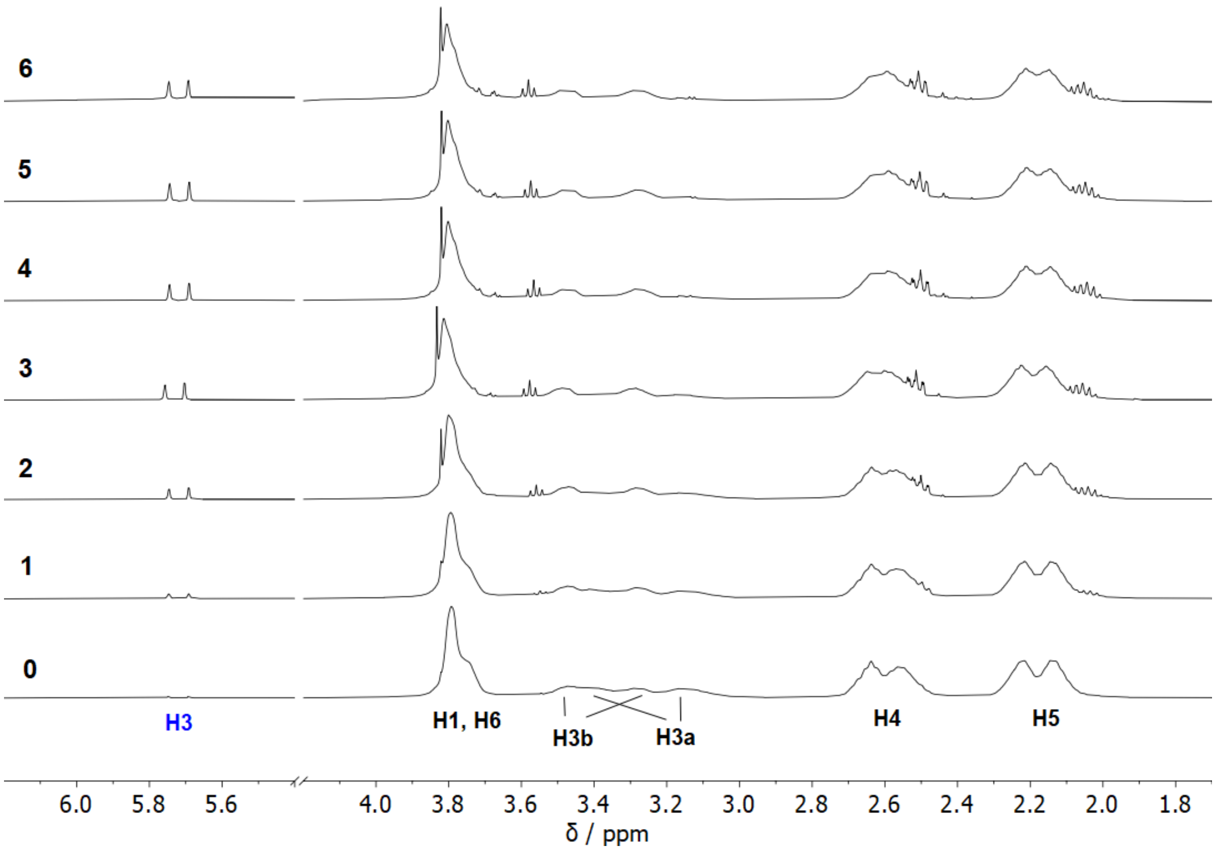


**Figure S1:** Kinetic study by $^{1}H$ NMR spectroscopy (400 MHz, 90% $H_2O$, 10% $D_2O$, 8.5 pH) on ultrasmall silver nanoparticles, stored at 4 °C dispersed in water. The particles were analyzed immediately after synthesis (0) and after one to six weeks in dispersion (1-6).

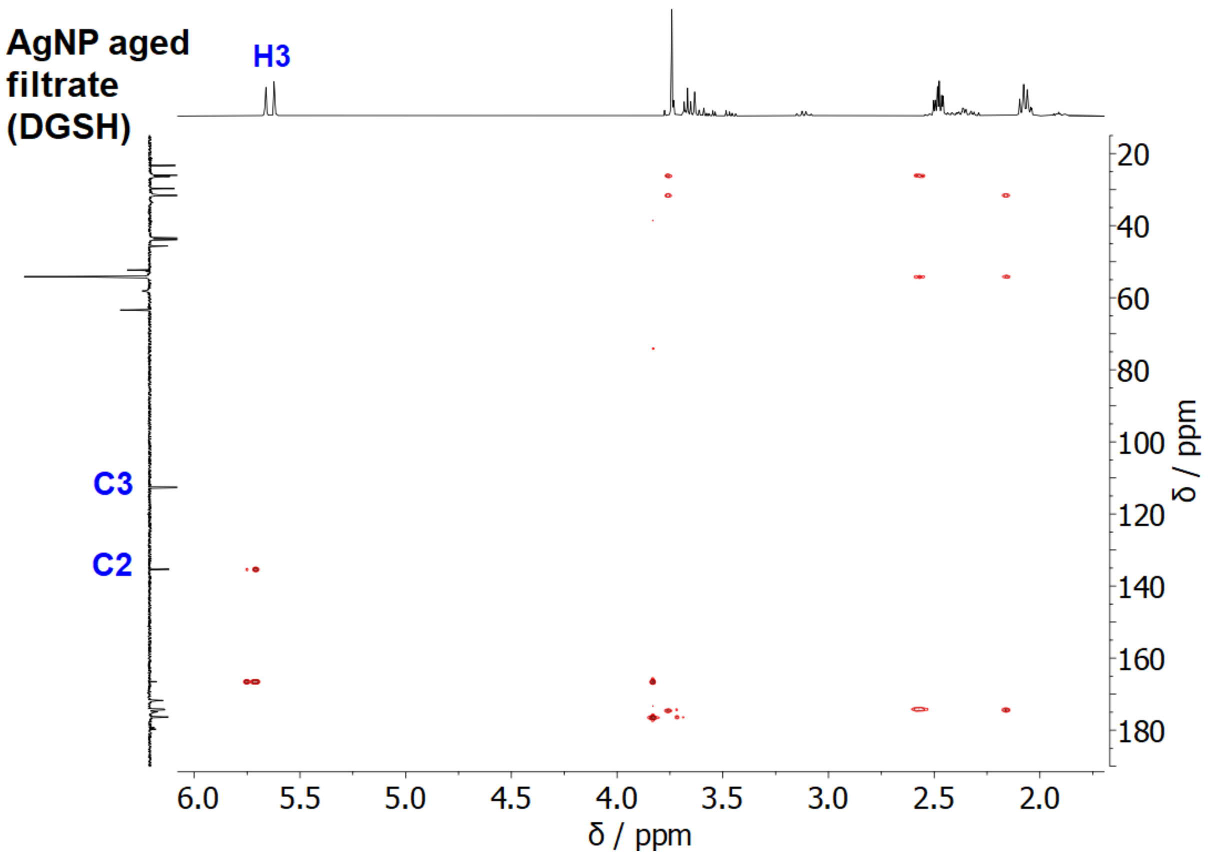


**Figure S2:** $^{1}$H-$^{13}$C HMBC NMR spectrum of aged silver nanoparticles (600 MHz, 90% $H_2O$, 10% $D_2O$; pH 8.5).
